# Chicago Society of Physicians and Surgeons

**Published:** 1878-03

**Authors:** Junius M. Hall


### CHICAGO SOCIETY OF PHYSICIANS AND SURGEONS.

REPORTED BY JUNIUS M. HALL, M. D.

Regular meeting at Grand Pacific Hotel, Jan. 28th, 1878, the President, Wm. H. Byford, M. D., in the chair.

At the last meeting of the society held at the rooms of the Chicago Medical Press Association, it was moved by Dr. Bevan, and unanimously passed by the society, that the next meeting be held at the Grand Pacific hotel. The secretary was directed to see Dr. W. Godfrey Dyas, and give him a special invitation to read his paper upon the lymphatic system at the next meeting. After the reading and acceptance of the minutes of the last meeting, Dr. Dyas was presented to the society by Dr. Byford, who remarked it was hardly necessary to introduce a gentleman, so long a resident in Chicago, and one so well known to the profession. The paper read was the first one of a series of four which the Doctor has been a number of years preparing, and was exclusively devoted to the anatomy of the lymphatic system. Frequent reference was made to the French and German investigators, but by far the most confidence was placed in the investigations of the great French anatomist, Sappey.

Eristratus (under the Ptolomies in Egypt) was the first discoverer of the chyliferous vessels, in the vivisection of kids, and mistook them for arteries.

Subsequently another anatomist traced them from the intestine to their termination in the mesenteric gland, and concluded they belonged to the veinous system.

Until the early part of the 17th century, the lacteal vessels were supposed to pass directly to the liver, but at this time they were found to pass uninfluenced by the liver until they reached the subclavian vein. John Hunter concluded from experiments, that this system *exclusively* presided over absorption, and this was the prevailing opinion until Magendie in France, and Tiedemann in Germany, proved that the veinous system participated.

In regard to the origin of the lymphatics, he groups the opinions under three heads.

1st. Lymphatics are closed at their origin.

2d. They are not closed, but communicate with the corpuscles of the connective tissue.

3d. They are not closed, but communicate with what Recklinghausen calls the plasmatic channels.

These vessels must pass through a ganglion or lymphatic gland, "a necessary condition of every lymphatic vessel." In man, it is impossible for a lymphatic vessel to reach the general circulation without passing through at least one gland.

Recklinghausen places these connective tissue corpuscles in their relation to the lymphatics, in the lacunæ or slits of the fibrillary substance of the connective tissue. These lacunæ are stellate spaces, the points anastomosing with each other by means of minute capillaries, which form loops, and also anastomose with the sanguineous system of capillaries, and thus communicate with the general circulation. The diameter of these capillaries is such, that nothing can pass through them from the blood vessels but serum.

*Formation of white blood globules.* The lacunæ are found filled with very fine granulations, rudiments of future white blood corpuscles, which are carried along into the larger lymphatic vessels. In contradiction of the statement made by some authorities, that the glands alone are the special organs for the formation of these organs, he states the following:

1st. White blood globules are found in the blood of some of the vertebrata that possess a lymphatic system, but no glands.

2d. White blood globules or leucocytes may be seen in the lymphatic system, at the distal end before they reach any gland.

"We are all aware that the lymphatic system terminates in "two trunks. The thoracic duct, which commences at the second

"lumbar vertebra, passes to the left side of the neck, forming "there an arch, and opens into the left subclavian vein, where it "unites with the internal jugular. The other, the great right "lymphatic vein, terminates in the right subclavian, where this "unites with the jugular vein."

Do the lymphatic trunks communicate with the veinous system elsewhere? Do the lymphatic capillaries and the sanguineous capillaries unite in the glands?

In the absence of positive knowledge we must take facts from comparative anatomy. As we ascend in the scale of the vertebrata, the communication between the lacteal vessels and the veins becomes more and more restricted. When we reach the mammalia, we find the following characteristics assigned them.

1st. Greater development of valves.

2d. Distinction of the lymphatics into two layers, superficial and deep-seated.

3d. A considerable number of ganglia and more limited communication with the veinous system.

"At the present time few anatomists maintain the communica-"tion in man of a lymphatic trunk elsewhere than at the junction "of the subclavian and internal jugular veins."

Do the two systems communicate in the glands? Tiedemann and Fohrmann think so for the following reasons: "The marked "contrast between the number and size of the lacteal vessels and "the small caliber of the thoracic duct. The continuation of life "when the duct has been obliterated. The passage of chyle into "the sanguineous system when the mesenteric glands are affected "by tubercularization provided those nearest the intestinal canal "are unaffected by disease."

Some authorities claim they can inject the veinous capillaries in the glands from the afferent vessels. Cruveilhier, Carpenter and Sappey deny this. "When the injection thus pushed is seen "in the veins, it is the result of a rupture which easily takes place "when the gland is softened from commencing decomposition, "but in every instance where injection was made in a gland fresh "and free from decomposition, the efferent vessels have been filled, "but not the veinous capillaries."

Prof. Nelson spoke of the relation of the lymphatic capillaries to the epithelium and sanguineous capillaries, a fact which had hitherto escaped his attention. Where you find the pavement epithelium, you have the lymphatic capillaries directly beneath, and below them, the vascular network. This is invariably the rule, with the exception of the mucous membrane of the lungs. Where you find the columnar epithelium, you have the vascular network directly beneath, and then the lymphatic vessels.

How this relationship influences disease, Dr. Dyas will show at some subsequent meeting.

A vote of thanks was given Dr. Dyas for his very interesting paper, and the Society hoped he would again favor them at some future meeting.

Dr. Byford spoke of the pleasure it gave him to see so large a number present, and then referred to the recent small attendance and lack of interest manifested by the members of the Society. Thinking it might be due to the change of the place of meeting, he asked for an opinion of the Society in regard to it.

Dr. Hamill said he had no hesitation in saying he had been deterred from attending the meetings because he did not feel able to climb the stairs necessary to get to the rooms of the Press Association. He moved that the meetings of the Society he held hereafter at the Grand Pacific Hotel.

Unanimously passed by the Society.

The Society then adjourned.

Regular meeting, Monday evening, February 11th, 1878.

Dr. Bevan in the chair.

The Secretary read the minutes of the last meeting, which were accepted by the Society.

Prof. D. T. Nelson was admitted as a member of the Society, by a unanimous vote.

The remainder of the evening was devoted to the report of the section upon nervous and mental diseases.

The first paper read was by Dr. P. S. Hayes, and consisted of a condensed abstract of recent investigations and experiments upon the pneumogastric nerves, by foreign investigators.

M. Tripier, in comparing the action of the right and left pnuemogastrics, says the right pneumogastric appeared to act more especially on the heart and the left on the lungs, but with some variation, according to the species of the animals. Section of one of the pneumogastrics would cause death. Two cases of death in man, after division of the right pneumogastric, are on record.

Prof. Langendorff observes that the influence by which reflex actions are exhibited may start either in the brain or in the periphery. The cerebral form is the function of a certain part of the brain, and during life is in a constant condition of mean tension. Experiments show that this function is not confined to the lobi optici, but that the cerebral hemispheres are implicated, and that the inhibitory reflex power of the brain is an essential factor of the intelligence.

Prof. F. Lusanna and F. Ciotto give the results after dividing both pneumogastrics in a dog, the animal living eighteen days. No signs of suffocation (respiration slow and deep) indicating that the vagi are not necessary for the maintenance of respiration, although modifying respiratory movements. No hyperæmia nor atelectasis, indicating that the vasomotor supply of the bronchi is, at least partly, sympathetic. Heart's action accelerated. Digestion affected chemically and mechanically. Œsophagus and stomach paralyzed.

M. Onimus says an ordinary Faradic current will be interrupted about the same number of times nervous shocks can be transmitted along special nerves. Striped muscles usually respond well to the stimulus, but the automatic and rhythmically acting organs require some numerical relation to be observed between their rhythm and the stimulus, or the effect will be perturbation instead of increase of function.

If this should prove true, it may be a very valuable and efficient mode of applying electricity in case of chloroform poisoning.

Dr. H. M. Bannister gave, with some general remarks on the disputed points in regard to the functions of the brain, a résumé of some researches by MM. Pétres and François Franck, and remarked on their bearings on the questions in dispute.

Dr. Charles E. Davis reported three cases of insanity occurring in private practice. One of them, a very interesting case, followed gunshot wound of the sympathetic in the neck.

Dr. Curtis presented to the society an intestinal calculus, phosphatic, about the size of an ordinary bean, recently removed from a young man who had died from typhlitis. He followed with some remarks upon the rarity of the affection in man, and its frequency in some species of animals.

The meeting then adjourned.

